# Priming With Red Blood Cells Allows Red Blood Cell Exchange for Sickle Cell Disease in Low-Weight Children

**DOI:** 10.3389/fmed.2021.743483

**Published:** 2021-12-22

**Authors:** Olivier Hequet, Camille Boisson, Philippe Joly, Daniela Revesz, Kamila Kebaili, Alexandra Gauthier, Celine Renoux, Severine Creppy, Elie Nader, Jean François Nicolas, Frédéric Berard, Fabrice Cognasse, Marc Vocanson, Yves Bertrand, Philippe Connes

**Affiliations:** ^1^Etablissement Français du Sang Rhône Alpes, Apheresis Unit, Centre Hospitalier Lyon Sud, Lyon, France; ^2^CIRI, International Center for Infectiology Research, INSERM U1111, Université de Lyon, Lyon, France; ^3^Laboratoire Interuniversitaire de Biologie de la Motricité (LIBM) EA7424, Equipe “Biologie Vasculaire et du Globule Rouge”, Université Claude Bernard Lyon 1, Lyon, France; ^4^Laboratoire d'Excellence Sur le Globule Rouge (Labex GR-Ex), Paris, France; ^5^Service de Biochimie et Biologie Moléculaire, Laboratoire de Biologie Médicale Multi-site, Hospices Civils de Lyon, Lyon, France; ^6^Institut d'Hématologie et d'Oncologie Pédiatrique, Hospices Civils de Lyon, Lyon, France; ^7^Distribution Unit, Centre Hospitalier Edouard Herriot, Etablissement Français du Sang Auvergne Rhône Alpes, Lyon, France; ^8^Clinical Immunology and Allergology, Centre Hospitalier Lyon Sud, Hospices Civils de Lyon, Lyon, France; ^9^Scientific Department, Etablissement Français du Sang Auvergne-Rhône-Alpes, Saint-Etienne, France

**Keywords:** red blood cell exchange, sickle cell anemia, low weight children, priming, safety, performances, blood rheology

## Abstract

Red blood cell exchanges are frequently used to treat and prevent cerebrovascular complications in patients with sickle cell anemia (SCA). However, the low weight of young children represents serious concerns for this procedure. The Spectra Optia device can perform automatic priming using red blood cells (RBCs) (RCE/RBC-primed) which could allow RBC exchanges (RCE) to be performed in young children without hypovolemic complications, but this method requires evaluation. We prospectively analyzed the clinical safety of the RCE/RBC-primed procedure in 12 SCA low-weight children under either a chronic RCE program or emergency treatment over 65 sessions. We monitored grade 2 adverse events (AEs) such as a decrease in blood pressure, increase in heart rate, fainting sensation, or transfusion reactions and identified the critical times during the sessions in which AEs could occur. Post-apheresis hematocrit (Hct) and a fraction of cell remaining (FCR) values were compared to the expected values. We also compared the impact of automatic RCE (*n* = 7) vs. RCE/RBC-primed (*n* = 8) on blood viscosity and RBC rheology. A low incidence of complications was observed in the 65 RCE sessions with only seven episodes of transient grade 2 AEs. Post-apheresis Hct and FCR reached expected values with the RCE/RBC-primed method. Both the automatic and priming procedures improved RBC deformability and decreased the sickling tendency during deoxygenation. Blood rheological features improved in both RCE/RBC-primed and automatic RCE without priming conditions. The RCE/RBC-primed procedure provides blood rheological benefits, and is safe and efficient to treat, notably in young children with SCA in prophylactic programs or curatively when a SCA complication occurs.

## Introduction

Sickle cell anemia is caused by a point mutation in the seventh position of the β-globin gene leading to the production of abnormal hemoglobin called HbS. When deoxygenated, polymerization of HbS may occur, leading to mechanical distortion of red blood cells (RBC), i.e., sickling (1). Decreased deformability and increased fragility of sickled RBCs are the cause of frequent painful vaso-occlusive events and enhanced hemolysis in patients with sickle cell anemia (SCA), respectively (1, 2). Moreover, patients with the highest blood viscosity are at high risk for frequent vaso-occlusive crises and other complications (2, 3). Recurrent vaso-occlusive events additionally cause endothelial dysfunction and inflammation, resulting in progressive tissue and organ damage (3–5).

Replacement of sickle RBCs containing HbS with healthy RBCs containing normal hemoglobin improves the outcome of patients with SCA. This replacement can be achieved by transfusions associated with manual bloodletting. However, this methodology allows a limited exchange of abnormal RBCs and may generate blood hyperviscosity, which in turn may result in vascular complications, and does not prevent iron overload (6). RBC exchange (RCE) using an apheresis device can replace a greater number of RBCs, is more efficient in preventing the occurrence of SCA complications when regularly performed, decreases whole blood viscosity, and limits the risk of iron overload (6–10).

However, the extracorporeal volume (EV) (160–185 ml depending on the device) of the RCE circuit limits its use in low-weight children (11–13). Transfusion experts suggest performing RCE without priming only in children with a bodyweight higher than 20 kg (with total blood volume [TBV] around 1,500 ml and EV corresponding to 12%) (14). When EV is greater than 15% of the total blood volume (TBV), they recommend performing blood priming before RCE (15). In addition to weight, hematocrit (Hct) must be considered when performing RCE since low weight associated with low Hct predisposes to hypovolemic complications during RCE (16).

A few years ago, the Cobe Spectra^®^ device was replaced by Spectra Optia^®^, which was the first device relying on a completely automatic priming procedure using RBC during RCE (RCE/RBC-primed). RCE/RBC-primed could allow RCE to be performed in young children without hypovolemic complications but this method requires evaluation. The main goal of this study was to assess the feasibility and safety of RCE/RBC-primed in low-weight children with SCA. In addition, we evaluated the impact of RCE/RBC-primed on RBC rheology and blood viscosity and compared the responses to the ones obtained with RCE without RBC priming.

## Materials and Methods

### Patient Selection

This prospective study was conducted in accordance with the Declaration of Helsinki and approved by the Hospices Civils de Lyon ethics committee (L14-127). Twelve children with SCA were included after obtaining parental written informed consent. The inclusion criterion was a body weight < 20 kg and the exclusion criterion was hemodynamic instability with mean systolic blood pressure (sBP) < 50 mmHg.

### Description of the RCE With Personalized Priming Procedure

In chronic programs, RCE sessions were performed every 6 weeks. Otherwise, RCE sessions were performed when an acute SCA complication occurred. Venous access was short-term central venous access in all cases.

Irregular agglutinin research was performed before RCE sessions. Transfused RBC units (RBCUs) were sickle-negative, leuco-reduced, crossmatch-compatible, and phenotypically matched for the C, E, c, e, and K (JK2 and MNS3 when possible) antigens. All the RBCUs transfused were heated to 37°C in a water bath.

Considering the low weight of the children, the TBV was calculated using the initial and simplified formulae of Glicher: TBV (ml) = weight (kg) × 75 (17). The number of RBCUs needed can easily be deduced since the Spectra Optia^®^ software (version 7.2) calculates the volume of transfused RBCs required to reach the predictive fraction of cell remaining (pFCR) and the predictive Hct (pHct). To perform RBC priming, a supplementary compatible RBCU (called reconstituted RBCU) was ordered and completed with ABO-compatible fresh frozen plasma to obtain a reconstituted RBCU. To prevent hemoconcentration during the critical session stage, i.e., after infusion of 185 ml of concentrated RBCs while the device has not removed significant amounts of abnormal RBCs, we primed the circuit with RBCU diluted with ABO-compatible fresh frozen plasma targeting an Hct close to the blood level of the patient, i.e., with an Hct of 28%, based on our clinical experience.

After discontinuation of the priming circuit (volume 200 ml and flow 60 ml/min), the reconstituted RBCU was removed, and the RBC-primed circuit was connected to the central venous access. Depending on the degree of filling of the infusion chamber, the EV of the RCE for the Spectra Optia^®^ (Terumo BCT) device circuit ranges from 141 to 185 ml (14), and the latter value was chosen for further calculations. Since rinse-back is not recommended at the end of the RCE in children (15), 15 ml/kg of 4% albumin was continuously perfused to prevent hypovolemia at the end of the procedure.

Anticoagulation of the circuit was performed using acid citrate dextrose solution A (ACD-A), and a significant decrease in blood calcium concentration was prevented by continuous intravenous calcium infusion.

### Clinical Tolerance, a Fraction of Cell Remaining, Hematocrit, and Factors Prone to Influence Hemostasis

The clinical tolerance of RCE using RCE/RBC-primed in low-weight children was divided into three periods: during the first 10 min of the RCE/RBC-primed sessions, corresponding to the time between the blood intake of the patient and the infusion of normal RBCs (called the critical time of priming reinfusion), and during two consecutive 40-min periods. Systolic BP and heart rate (HR) were monitored every 2 min during the critical time of priming reinfusion and every 10 min during the remaining session. According to the National Cancer Institute Common Terminology Criteria for Adverse Events guidelines (18), grade 2 AEs or higher correspond to a significant decrease in sBP (>20 mmHg) and a significant increase in HR (>20 pulses per minute) associated or not with a significant fainting/asthenia sensation. The number of RCE/RBC-primed sessions with grade 2 AEs or higher were recorded, as well as the symptoms of transfusion-related AEs (pruritus, urticarial erythematous wheals, skin edema, and wheezing).

At the end of the RCE sessions, post-apheresis Hct was assessed to obtain actual Hct (aHct). In parallel, the post-apheresis percentage of HbS (actual Hbs or aHbs) was assessed to calculate actual FCR (aFCR). The aFCR was calculated as follows: aFCR = aHbS)/pre-apheresis HbS (also called native HbS or nHbS). We also noted the predictive FCR (pFCR) which is recorded on the device before the sessions and the final FCR (fFCR), which corresponds to the FCR recorded by the device at the end of the sessions. In parallel, we noted pre-apheresis or native Hct (nHct), predictive Hct (pHct, recorded on the device before the session), and final Hct (fHct, recorded on the device at the end of the sessions).

Changes in factors prone to influence hemostasis parameters were assessed, i.e., ratio of ACD-A/calcium infused and decrease in platelet blood levels (calculated as follows: decrease in platelet level = [pre-apheresis blood level – post-apheresis blood level]/pre-apheresis blood level).

### Blood Rheology

**Figure 1:**
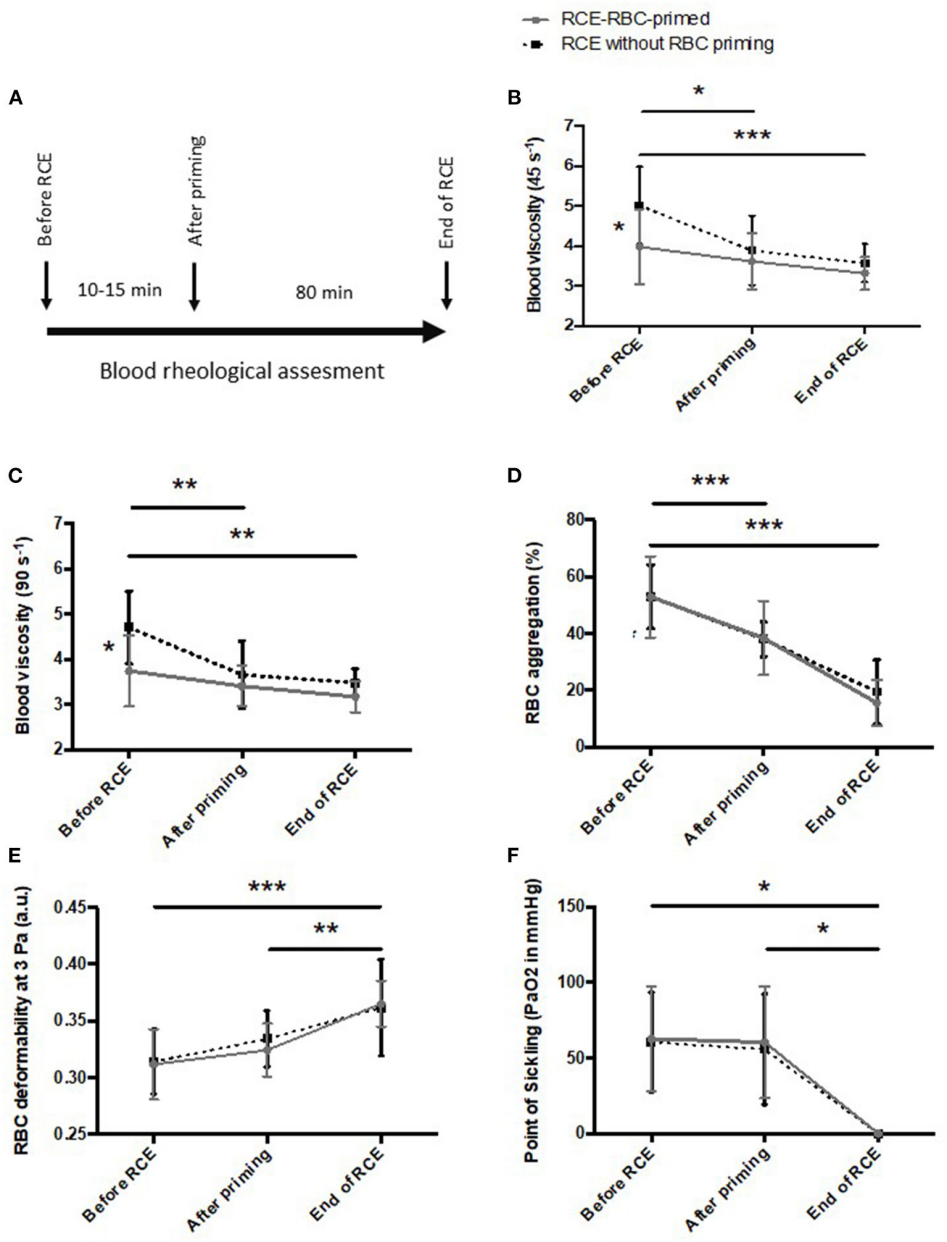
Changes in blood rheological properties during red blood cell exchange/red blood cell (RCE/RBC)-primed vs. RCE without priming. **(A)** protocol; **(B)** blood viscosity at 45 s^−1^; **(C)** blood viscosity at 90 s^−1^; **(D)** RBC aggregation; **(E)** RBC deformability at 3 Pa; **(F)** Point of sickling. Statistical difference: *p < 0.05; **p < 0.01; ***p < 0.001.

We also analyzed the impact of RBC-priming by comparing RCE/RBC-primed (*n* = 8 sessions in 5 of the 12 patients followed) vs. automatic RCE (i.e., without priming RBCs; *n* = 7 sessions in 7 other patients) on blood viscosity and RBC rheology. The two groups of patients were assessed during RCE programs. Measurements were performed before RCE/RBC-primed, at the end of the critical time of priming reinfusion (10–15 first min) and the end of the sessions (Figure 1A). The same blood rheological parameters were measured at the same time points in patients having the RCE/RBC-primed and automatic classical RCE. Blood viscosity was measured at native Hct and shear rates of 45 and 90 s^−1^ using a cone-plate viscometer (Brookfield DVII+ with CPE40 spindle, Brookfield Engineering Labs, Natick, MA, USA) and expressed in centipoises (cP). RBC deformability was determined at 37°C, in isotonic conditions and 3 Pa by laser diffraction analysis (ektacytometry), using the Laser-assisted Optical Rotational Cell Analyzer (LORRCA Maxsis, RR Mechatronics, Hoorn, The Netherlands). In addition, ektacytometry was carried out with the oxygenscan module (LORRCA Maxsis, RR Mechatronics, Hoorn, The Netherlands) to measure RBC deformability over an oxygen gradient as previously described (19–22). The suspension was sheared at 30 Pa and 37°C into the Couette system of the ektacytometer. The oxygen partial pressure (pO_2_) was gradually decreased from 160 to 20 mmHg (deoxygenation) and then increased to normoxic values. The diffraction pattern obtained by ektacytometry was analyzed with a camera and a computer and the elongation index (EI), which reflects RBC deformability, was also calculated. The Point of Sickling (PoS) was obtained during deoxygenation and corresponds to the pO_2_ at which RBC deformability decreases below 5% of the maximal deformability reached during normoxia (i.e., before deoxygenation). All measurements were standardized as recommended (21, 22). RBC aggregation was determined at 37°C *via* syllectometry, (i.e., laser backscatter vs. time, using the LORRCA) after adjustment of the Hct to 40%.

### Statistical Analyses

Values are expressed as mean ± *SD*. A one-way ANOVA with repeated measurements was used to compare aHct, pHct, and fHCT; aFCR, pFCR, and fFCR. A two way-ANOVA was used to compare the effects of RCE/RBC-primed and RCE without RBC priming on blood rheology. The Tukey *post-hoc* test was used to locate differences when appropriate. *p* < 0.05 was considered significant.

## Results

### Feasibility and Safety of the Priming Protocol and the Whole RCE

Twelve children were treated with RCE/RBC-primed (Table 1). In most children (10/12), EV of the circuit represented a high volume related to their TBV (EV/TBV ratio greater than 15%) while lower EV/TBV was associated with low nHct (21 and 22% for patients 6 and 12, respectively) were found in two of them.

**Table 1:** Clinical characteristics of the 12 patients with sickle cell anemia (SCA) at the beginning of red blood cell exchange/red blood cell (RCE/RBC)-primed.

**Parameters**	**Pt 1**	**Pt 2**	**Pt 3**	**Pt 4**	**Pt 5**	**Pt 6**	**Pt 7**	**Pt 8**	**Pt 9**	**Pt 10**	**Pt 11**	**Pt 12**
Gender	F	F	F	M	M	M	F	M	F	M	F	F
Age (year)	3	3.5	3.5	3	3	6	3	4.5	4.5	3	5.5	6
Weight (kg)	11	15	13	12	13	18	13	16	17	13	17	19
TBV (mL)	825	1,050	975	900	975	1,350	975	1,200	1,190	952	1,270	1,425
EC/TBV (%)	22	17	19	20	19	14	19	15	16	19	15	13
nHct (%)	28	31	21	28	26	21	22	23	12	30	22	22
RCE Program	Yes	yes	yes	no	yes	yes	yes	no	no	no	no	yes
Indications for RCE	ACS+ Stroke	ACS	ACS+ VOC	ACS	ACS+ VOC	CVO+ Surgery	Stroke	ACS	Meningitis	Surgery	Surgery	Cerebral vasculopathy
nHbS (%)	31	46	84	81	83	75	88	84	92	61	87	91

*Pt, patient; F, female; M, male; TBV, Total blood volume; VOC, vaso-occlusive crisis; ACS, acute chest syndrome; RBCP, packed red blood cells; RCE, red blood cell exchange; nHct, native hematocrit (blood hematocrit before RCE); nHbs, native HbS (blood HbS before RCE)*.

During all sessions, it took approximately ten min (9 to 15 min) for the device to reinfuse the volume of RBC-primed while the blood of the patient was still being removed. No significant grade 2 AEs (no decrease in sBP, no increase in HR, no fainting sensation) were observed during that time in the 65 RCE/RBC-primed sessions performed (Table 2).

**Table 2:** Clinical complications during RCE/RBC-primed.

**Parameters**	**Pt 1**	**Pt 2**	**Pt 3**	**Pt 4**	**Pt 5**	**Pt 6**	**Pt 7**	**Pt 8**	**Pt 9**	**Pt 10**	**Pt 11**	**Pt 12**	**All pts**
Number of RCE/RBC-primed sessions	28	10	8	2	5	3	4	1	1	1	1	1	65
Decrease in sBPS													
1^st^ period (0–10 min)	0	0	0	0	0	0	0	0	0	0	0	0	0
2^nd^ period (11–50 min)	0	0	0	0	0	0	0	0	0	0	0	0	0
3^rd^ period (51–90 min)	1 epd*	0	0	0	1 epd*	0	0	0	0	0	0	0	2
Increase in pulses													
1^st^ period (0–10 min)	0	0	0	0	0	0	0	0	0	0	0	0	0
2^nd^ period (11–50 min)	0	0	0	0	0	0	0	0	0	0	0	0	0
3^rd^ period (51–90 min)	0	1 epd	0	0	1 epd	0	0	0	0	0	0	0	2
Fainting sensation													
1^st^ period (0–10 min)	0	0	0	0	0	0	0	0	0	0	0	0	0
2^nd^ period (11–50 min)	0	0	0	0	0	0	0	0	0	0	0	0	0
3^rd^ period (51–90 min)	2 epd	0	0	0	0	0	1 epd	0	0	0	0	0	3
Transfusion reactions (0–90 min)	0	0	0	0	0	0	0	0	0	0	0	0	0

Pt, patient; sBP, systolic blood pressure; epd, episode. *occurrence during 5 min after the end of the sessions.

The mean pFCR was 19 ± 4% (Table 3), which corresponded to a significant RBC replacement (68 ± 8 ml/kg) of at least 80% of the initial RBC volume. As recommended (15), the target Hct after RCEs in children was expected to be 27–30%. Mean Hct levels in children after RCEs (aHct) were not significantly higher than nHct (27 ± 2 vs. 26 ± 3%, respectively), reducing the risk for developing hemodynamic complications during and after RCEs (Table 3).

**Table 3:** Biological results to assess performances of RCE/RBC-primed.

**Parameters**	**Pt 1**	**Pt 2**	**Pt 3**	**Pt 4**	**Pt 5**	**Pt 6**	**Pt 7**	**Pt 8**	**Pt 9**	**Pt 10**	**Pt 11**	**Pt 12**	**All pts**
nHct (%)	28 ± 1	25 ± 3	22 ± 1	26 ± 2	26 ± 3	26 ± 5	23 ± 1	23	12	30	30	12	26 ± 3
pHct (%)	27 ± 1	27 ± 1	26 ± 1	27 ± 1	27 ± 1	27 ± 1	26 ± 1	28	25	29	28	26	27 ± 1
fHct (%)	28 ± 1	27 ± 5	25 ± 1	26 ± 2	27 ± 1	29 ± 1	26 ± 1	25	25	29	30	26	27 ± 1
aHct (%)	27 ± 2	28 ± 3	26 ± 2	27 ± 1	28 ± 1	29 ± 1	25 ± 1	25	26	29	29	26	27 ± 2
pFCR (%)	17 ± 4	18 ± 3	19 ± 5	17 ± 3	18 ± 3	20 ± 0	21 ± 2	20	20	20	18	20	19 ± 4
fFCR (%)	16 ± 4	16 ± 2	17 ± 7	17 ± 4	17 ± 5	19 ± 0	23 ± 2	20	20	20	19	26	18 ± 5
aFCR (%)	14 ± 6	17 ± 4	19 ± 7	12 ± 2	15 ± 6	17 ± 6	23 ± 4	19	23	19	18	22	17 ± 6
Number of RBCUs/session	3 to 4	3	2 to 3	3	2 to 3	3 to 4	2 to 3	2	3	2	3	3	2 to 4
ACD-A infused to Pt (mL)	116 ± 22	101 ± 8	94 ± 18	91 ± 10	99 ± 11	108 ± 12	87 ± 19	124	44	88	103	152	106 ± 22
Calcium injected (mL)	11 ± 3	13 ± 3	10 ± 3	9 ± 0	10 ± 3	15 ± 3	10 ± 2	11	9	11	16	15	11 ± 3
Ratio ACD-A/calcium used	11 ± 2	9 ± 2	10 ± 1	11 ± 1	10 ± 3	7 ± 1	8 ± 1	11	5	8	6	10	10 ± 2
PBL before RCE/RBC-primed	183 ± 62	252 ± 62	304 ± 50	432 ± 179	297 ± 98	394± 154	398 ± 53	151	186	269	353	304	277± 103
PBL after RCE/RBC-primed	79 ± 13	79 ± 24	75 ± 12	92 ± 15	54 ± 27	210 ± 57	152 ± 48	69	106	119	150	167	97 ± 46
Decrease in PBL (%)	52 ± 10	69 ± 11	76 ± 4	76 ± 14	79 ± 3	56 ± 15	61 ± 14	54	43	56	57	45	61 ± 13

*nHct, native (pre-apheresis Hct); pHCt, predictive Hct (expected Hct registered on device before session); fHct, post-apheresis Hct given by device after session; aHct, actual Hct (post-apheresis peripheral blood Hct); pFCR, predictive FCR; fFCR, final FCR (expected FCR given by device after session); aFCR, actual FCR (post-apheresis Hbs divided by pre-apheresis HbS); Pt, patient; PBL, platelet blood levels*.

The exchange step lasted 80 ± 11 min. All complications occurred during the last 30 min or in the first 5 min after the end of the session (Table 2). Systolic blood pressure (sBP) decreased in two patients just after the end of the sessions (Table 2). One episode of a significant increase in heart rate occurred in two patients and a fainting sensation occurred during three sessions in two children, all during the last 30 min. Saline and 4% albumin were infused, inducing a decrease in HR or fainting sensation (while RCE continued) and a rapid increase in sBP. All the hemodynamic complications occurred at the end of the RCE sessions. The occurrence of AEs at this time suggested a role of the large volume of RBC exchanged (more than 80% of RBC exchanged i.e., aFCR < 20%) and we could speculate that exchanging lower amounts of RBC may decrease the incidence of AEs. No pruritus or urticarial wheals and no wheezing were observed thereafter during the entire protocol (Table 2).

### Performances of RCE/RBC-Primed

We analyzed the ability of the RCE/RBC-primed method to obtain the Hct required, and the ability to replace sufficient amounts of RBC in the blood of patients. No difference was observed between aHct, pHct, and fHct, or aFCR, pFCR, and fFCR (Table 3), indicating that the expected Hct and FCR values are reached.

The ratio ACD-A/calcium infused was around 10 in most of the patients (Table 3), as observed in our experience in adults treated with RCE (data not shown). The mean decrease in platelet levels was 61 ± 13%, which needs to be considered in some situations when RCE is performed.

### Blood Viscosity and RBC Rheology: Comparison of RCE/RBC-Primed vs. RCE Without Priming

The use of an RBCU during the priming raised the question of transient blood hyperviscosity during the priming and at the end of the sessions that could promote complications in SCA. Indeed, blood rheological parameters were investigated in 8 young low-weight children with SCA (19 ± 1 kg; 4 ± 1.2 years) having the RCE/RBC-primed procedure and compared to the blood rheological responses of seven older children with SCA (33 ± 13 kg; 12.4 ± 3.6 years) who were having the classical automatic RCE procedure without RBC-priming (Figure 1). Of note, the children of both groups were under chronic RCE programs and none of the sessions assessed was performed in an emergency.

Initial blood viscosity was lower in the RCE-RBC-primed group compared to the group having RCE without RBC priming. Blood viscosity remained unchanged in the patients having RCE/RBC-primed (Figures 1B,C). However, during automatic (classic) RCE, blood viscosity decreased after the first RBCU to reach similar blood viscosity to the other group up until the end of the procedure. RBC deformability increased and RBC aggregation decreased in the two groups over the procedures, with no difference between them (Figures 1D,E). The PoS decreased in the two groups to reach very low values at the end of the procedure and no difference was observed between the two groups (Figure 1F).

## Discussion

The indications of RCE during curative and prophylactic treatments of SCA are well-known (23). The principle of RCE is to prevent the occurrence of SCA complications by replacing abnormal RBCs with normal RBCs so that the HbS level does not exceed a defined threshold between two sessions (15, 23). Since prophylactic treatment has become more routine among apheresis teams who treat more and more Patients with SCA, the RCE method has to be improved to optimize this treatment in all patients, including the youngest ones. In very young children, two main issues hamper the use of RCE on a routine basis: 1) the venous access series [this is why we used temporary central venous catheter access for which we demonstrated tolerance and efficiency (24)] and 2) the risk of hypovolemia. The availability of a device combining RCE and an automatic priming step (RCE/RBC-primed) led us to evaluate its feasibility, safety, and performance in this population.

Our work showed that the RCE/RBC-primed method was well tolerated both during the priming and during the RCE step itself. The incidence of complications was thus very low and rapidly self-limiting. The method was safe without any risk of increasing blood viscosity during and after the sessions. Moreover, the RCE/RBC-primed method was efficient. i.e., allowed to reach the targeted post-apheresis FCR and Hct.

The main problem when performing RCE in very young children is the risk of hypovolemia due to a high EV compared to a low TBV. Indeed, the study of Dedeken et al. switched from manual exchange transfusion to RCE (using the Optia Spectra system) only in children who weighed 30 kg or more, this threshold having been decided to avoid priming of the circuit (7). RCE techniques with low EV, such as COBE Spectra, were used in low-weight children (20 to 30 kg) without inducing hemodynamic complications (25, 26). In other apheresis techniques, RBC-priming appears to be a therapeutic option to treat children with a bodyweight below 20 kg (27). As mentioned, in our series the EV represented around 20% of the TBV of each child, which could induce severe hypovolemia and hemodynamic complications. Monitoring of the early phase of RCE while infusing the volume in the circuit primed with reconstituted RBCs showed no hemodynamic changes during initial blood withdrawal. This absence of early hemodynamic complications leads us to suggest that RBC-priming may be used more systematically in low-weight children. The complications that occurred in 10% of the sessions involved only hemodynamic events. Since they occurred at the end of the sessions, they were probably the consequence of extended RBC exchanges. Moreover, the occurrence of hemodynamic complications led us to consider the use of a continuous albumin infusion during RCE. However, this procedure with RBC-priming seemed to be necessary, as the frequency of AEs would probably be higher without RBC-priming in these low-weight children (14).

The performances of all types of RCE sessions, and here of RCE/RBC, had to be assessed to verify the efficiency and safety of this treatment in low-weight children. The main objective of RCE is to replace abnormal SCA-RBC with non-sickle RBC, which was evaluated by the FCR; the lower the FCR, the higher the replacement of SCA-RBC. Our results showed that the RCE/RBC-primed procedure was efficient since fFCR was similar to aFCR. Moreover, aHct was similar to both fHct and pHCt, excluding an increased risk for post-RCE stroke. Manual RBC exchange can cause a rise in Hct without improvement in blood viscosity (10, 28). The history of the first patient of this series summarizes the outcome and the rheological conditions after transfusion or manual RBC exchange and RCE. Within 24h after a transfusion for ACS, this child developed a stroke and was successfully treated by RCE. Single transfusion may increase Hct and blood viscosity and cause vaso-occlusive complications (28, 29). More recently it has been clearly shown that automated RCE and manual exchange display opposite changes in post-exchange Hct and blood viscosity (10). Manual exchanges can cause a rise in Hct without any improvement in blood viscosity while automated RCE decreases blood viscosity without any increase in Hct (10). As we added RBC during the infusion of primed-RBCs, we analyzed various parameters of blood rheology during and at the end of RCE, showing no increase in blood viscosity at the beginning of the session and a similar decrease in blood viscosity both in RCE/RBC-priming and RCE without RBC priming. The increase in RBC deformability, the decrease of the tendency of RBC to sickle (normal RBCs do not sickle), and the decrease in RBC aggregation during both RCE procedures may explain why blood viscosity decreased in the two groups, demonstrating no risk of blood hyperviscosity for the priming step.

Other factors need to be analyzed, in particular, hemostasis parameters as another blood product is transfused; this priming could influence the blood changes after sessions. Additional analysis of biological parameters showed a 60% decrease in platelet levels after RCE/RBC-priming. This decrease was in accordance with previous studies investigating the effects of RCE (30, 31). However, particular caution must be paid when performing RCE in patients with very recent stroke or at risk of bleeding, because of liver failure for example. In this last clinical situation, ACD-A infusion must be decreased or discontinued (32). In other cases, ACD-A infusion induces a decrease in calcium blood levels, which needs to be counteracted by calcium infusion. This calcium supplementation is particularly necessary for RCE in which ACD-A infusion is significant. The ratio between ACD-A and calcium (both infused) must be monitored, particularly in low-weight children. Even if no guidelines have yet been developed to consider the usage of calcium according to ACD-A, the ratio between both infusions must be monitored to prevent a bleeding risk associated with a decrease in platelets, especially when RCE is performed after stroke. In our study, the ratio ACD-A infused/calcium infused was monitored and was in the same range in all sessions. In our experience in the field of RCE, the ACD-A/calcium ratio of 10 prevents the symptoms of hypocalcemia and the bleeding risk (data not shown).

In conclusion, the usage of RBC-priming in low-weight children appears necessary and safe when performing RCE. RBC-priming did not modify the performances of RCE. Altogether, these results should encourage apheresis and pediatric hematological teams to perform RCE in these low-weight children while considering many precautions. In patients with higher body weight (20–30 kg) or low body weight (15–20 kg) but low Hct, another possibility would be to perform albumin-priming. However, this therapeutic possibility has yet to be evaluated.

## Data Availability Statement

The raw data supporting the conclusions of this article will be made available by the authors, without undue reservation.

## Ethics Statement

The studies involving human participants were reviewed and approved by Hospices Civils de Lyon Ethics Commitee (L14-127). Written informed consent to participate in this study was provided by the participants' legal guardian/next of kin.

## Author Contributions

All authors listed have made a substantial, direct, and intellectual contribution to the work and approved it for publication.

## Conflict of Interest

The authors declare that the research was conducted in the absence of any commercial or financial relationships that could be construed as a potential conflict of interest.

## Publisher's Note

All claims expressed in this article are solely those of the authors and do not necessarily represent those of their affiliated organizations, or those of the publisher, the editors and the reviewers. Any product that may be evaluated in this article, or claim that may be made by its manufacturer, is not guaranteed or endorsed by the publisher.

## Abbreviations

RBC: Red blood cell
RCE: Red blood cell exchange
SCA: Sickle cell anemia
AE: Adverse events
Hct: Hematocrit
FCR: Fraction of cell remaining
EV: Extracorporeal volume
TBV: Total blood volume
sBP: systolic blood pressure
RBCU: Red blood cell units
aFCR: actual FCR
pFCR: predictive FCR
fFCR: final FCR
nHct: native Hct
pHct: predictive Hct
fHct: final Hct
PoS: Point of sickling
ACD-A: acid citrate dextrose A.
